# Prison Suicide in Comparison to Suicide Events in Forensic Psychiatric Hospitals in Germany

**DOI:** 10.3389/fpsyt.2018.00398

**Published:** 2018-08-28

**Authors:** Alexander Voulgaris, Nadine Kose, Norbert Konrad, Annette Opitz-Welke

**Affiliations:** ^1^Department of Psychiatry and Psychotherapy, Prison Hospital Berlin, Berlin, Germany; ^2^Institute of Forensic Psychiatry, Charité University Berlin, Berlin, Germany

**Keywords:** suicide events, prison, forensic psychiatry, schizophrenia, mental disorder, substance abuse disorder

## Abstract

**Background:** There is limited international as well as national research on suicide events in prisons and in forensic psychiatric hospitals. This retrospective study compares completed suicide events within these two high-risk populations in state institutions over a time period of 5 years from 2000 to 2004.

**Material and Methods:** Data was collected through a nationwide survey: all forensic psychiatric hospitals within Germany were contacted via postal mail and received a questionnaire concerning the suicide events from 2000 to 2004. All federal lands of Germany were similarly assessed by a survey endorsed by the respective federal ministries of justice. All prison institutions (100%) participated in the survey, while 84% (53 units) of the forensic psychiatric hospitals nationwide contributed. A comparative statistical analysis was conducted using Fisher's exact test or the Mann-Whitney U-test (age). A multivariate logistic regression analysis was done to assess adjusted effects. For the Kaplan-Meier analysis, the months until suicide were analyzed followed by a Cox-regression analysis.

**Results:** There was no statistically significant difference between the mean suicide rate in forensic psychiatric hospitals (123/100.000, 95% confidence interval: [0.00103, 0.00147]) and in the prison system (130/100.000, 95% confidence interval: [0.00109, 0.00154]). Patients who committed suicide in the forensic hospitals were, in comparison to the prison system, more likely to have committed a violent offense and have had a prior history of suicide attempts. The duration from admission into the institution to the suicide event was significantly shorter in the prison group. Also, younger people commited suicide earlier during their stay in a forensic psychiatric hospital or prison.

**Conclusions:** While the results suggest a necessity to optimize data collection in the prison system (prior suicide events and history of mental disorder), it is important to discuss the current discharge arrangements within the forensic hospitals.

## Introduction

All European legislations recognize the concept of criminal responsibility as a prerequisite for punishment. Most European countries require some degree of reduced responsibility for the crime committed for entry into the forensic psychiatric system, while offenders with full responsibility can be subject to a prison sentence. In the UK, access to forensic psychiatric care is determined only on the basis of the mental condition at the time of assessment (1). Regarding the duration of stay, most countries allow detention of mentally disordered offenders beyond the length of the prison sentence their offense would have attracted had they been imprisoned (Sampson et al., submitted). In Croatia, Portugal, and Italy, the time of psychiatric detention is limited to the prison sentence the individual would have received without a mental disorder. In Germany, the longer the detention in a forensic psychiatric hospital, the more important the considerations are regarding the proportionality of the patient's right to freedom against any risk he or she may pose (2).

Although specialized forensic institutions exist in many countries, most offenders with mental disorders are found in prison settings (1). The international literature suggests an increased prevalence of mental disorders in prison inmates (3–6). Fazel and Seewald found a pooled prevalence of psychosis of 3.6% in male prisoners (3.9% in female prisoners) and for major depression, the prevalence was 10.2% in male prisoners (14.1% in female prisoners) (3). The rates for comorbidity ranged from 20.4 to 43.5% in those with any mental disorder who had comorbid substance misuse (3). The study by Fazel and Danesh that included nearly 23,000 prisoners from 12 countries showed similar results for schizophrenia and depression. Personality disorders were detectable in 65% of the male and 42% of the female detainees (4). In a German study, 88.2% of the subjects in a prison in Bielefeld were diagnosed with at least one mental disorder (7). Similar research among 80 randomly selected Greek prisoners yielded a prevalence of mental disorders of 78.7% among the participants and of 37.5% each for anxiety disorder and antisocial personality disorder (8).

In correctional settings, suicide is often the single most common cause of death (9, 10). Suicide prevention and the treatment of mentally disordered people with a higher risk of committing suicide are central aspects of the clinical work for psychiatrists and psychotherapists (11). It is well known that suicidality is a multifactorial conditioned phenomenon with general risk factors being mental disorder, hopelessness, impulsivity, former suicide attempts, age, gender, ethnicity, relationship status, and a positive family history with suicide events (12). Males are more likely than females to die by suicide, and middle-aged adults as well as elderly people, especially elderly males, are described as high risk groups for suicide (10, 13, 14). While major depression is the most common mental disorder in the general population and most often associated with suicide risk (15), another group at higher risk consists of young schizophrenic patients (16, 17). According to the findings of a Swedish study group (18), patients with a schizophrenia who were once treated because of suicidality committed suicide more frequently in the course of time in comparison to patients with other mental disorders. Apart from psychiatric disorders, there are indications that troubling “life events” lead to an increase in suicide risk (19) and it seems comprehensible that imprisonment, as well as admittance into a forensic institution, may be considered as such a life event (1, 20).

Suicide rates per 100,000 prisoners have been found to range from 58 to 147 in a review of 12 studies from Western countries compared to figures of 16 to 31 in the general population (21) and prisoners with psychosis, depression or substance abuse disorder are at an even greater risk (22). In Europe, the rate of prison suicide events correlated with the number of mentally disordered prisoners (23), and in 72% of prison suicide events, mental disorders were found in the specific medical history (24). A more recent German study by Opitz-Welke et al. (25) identified a mean suicide rate of 105.8 per 100,000 in male prisoners and 54.7 per 100,000 in female prisoners, with specific risk factors being the special situation (imprisonment), the separation of loved ones, pre-trial detention, small prisons, a single cell/isolation, (expectance of) a long sentence, former suicide attempts, and the arrest for a violent crime (26).

In a comparative study, Otte et al. (27) demonstrated that the level of mental distress measured via the Symptom Checklist-90-Revised and Brief Symptom Inventory was as high in long-term detainees as in patients of a general psychiatric hospital and even higher than in patients of forensic psychiatric institutions. The lowest level of mental distress was described in short-term detainees.

Although it is known that over the course of time, the number of patients in the forensic psychiatric system has increased significantly not only in Germany (28, 29) but in many Western European countries (30), literature on suicide events in these institutions is still very limited compared to prison suicide.

## Aim of the study

This study compares completed suicide events in two high-risk groups: the group of prison inmates and of the patients in the forensic psychiatric institutions in Germany (2000–2004). Due to the immense lack of information on suicide events in forensic psychiatric hospitals we want to determine a suicide rate for this specific setting. In our opinion, it is important to understand the impact of institutionalization on suicidality in forensic hospitals as well as in the prison system.

Our first hypothesis is that the rate of completed suicide events in forensic psychiatric hospitals is higher than that in prison. All patients in these settings suffer from a mental disorder and are exposed to the same general and specific risk factors as the prisoners are. Furthermore, in Germany, the duration of stay in a forensic hospital is potentially unlimited in comparison to a prison sentence. Considering this, we also hypothesize that in forensic hospitals, the time from admission to suicide is longer than in the prison system. This could lead to a new direction in suicide prevention in forensic hospitals pointing to the duration of stay in comparison to prison suicide, where literature suggests a higher risk in the first weeks of admittance. In addition, our aim is to describe the potential differences and similarities between the two high-risk groups.

## Materials and methods

Forensic psychiatric hospitals in Germany consist of two departments. According to §63 StGB (penal code), the duration of stay depends on the treatment prognosis. If the patient is no longer “dangerous” to the public, the institutionalization may be suspended on probation. According to §64 StGB, offenders with a leading psychotropic drug dependence syndrome and sufficiently concrete therapeutic prospects are confined to special detoxification centers in forensic psychiatric hospitals. Here, the duration of stay is generally limited to 2 years. In this study, patients of both departments were included.

Data was collected through a nationwide survey: all forensic psychiatric hospitals within Germany were contacted via postal mail and received a questionnaire concerning the completed suicide events from 2000 to 2004 in their hospitals. Attempted suicide events were not considered in this study. The questionnaires were sent directly to the head of the department of each forensic hospital. They were then completed directly by the medical staff of the forensic hospital and sent back to us via postal mail. This questionnaire was not standardized, and it asked for specific information regarding the patients who committed suicide: gender, age, nationality, relationship status, date of admittance to the hospital, date of suicide, school degree, legal status (pre-trial or sentenced), type of offense, mental disorder, former detention, and former suicide events. The type of offense was categorized as violent offense and non-violent offense. Violent offenses included homicide, murder, manslaughter, aggravated battery, arson, rape, and sexual violence. Mental disorders were defined by the medical staff of the forensic hospitals, on the basis of the Tenth Revision of the International Classification of Diseases for the classification of mental and behavioral disorders.

The numbers of prison suicide events on all federal lands of Germany were assessed by a survey endorsed by the respective federal ministries of justice. Data on the prison suicide events was collected through the use of a specific questionnaire. The respective federal lands rated the questionnaires. Information was attained using the reports on exceptional events from the routine documentation (“Generalakten”). Official data on occupancy rates in both institutions on a yearly reference date was used as a basis for the calculations of the mean suicide rates (28, 31). For the calculation of the confidence intervals, the method suggested by Agresti-Coull was used (32). Comparative statistical analysis was conducted using Fisher's exact test or the Mann-Whitney U-test (age). A multivariate logistic regression analysis was done to assess adjusted effects for the dependent (prison and forensic group) and the independent variables (see Table 1). We added the confidence intervals for the estimated odds ratios of the variables that were found statistically significant. The variable mental disorder was underreported and not considered for the multivariate logistic regression analysis. For the Kaplan-Meier analysis, the time in months until the suicide events were analyzed. Censored cases did not occur. Cox regression models were subsequently defined with the time to suicide as the dependent variable and the two groups (forensic hospital, prison) as independent variables. For all analyses referring to the time until suicide, an adjustment for age was done. For that, the whole population was divided into two groups of equal size (median-split). All analyses were performed with the R statistical software, Version 3.5 and the “survival” and “survminer” packages.

**Table 1:** Suicide events in prisons and in forensic hospitals in Germany (univariate analysis).

	**Prison 2000–2004**			**Forensic hospital 2000-2004**			
Total		479			40		
Male		475	99%		37	93%	
Female		4	1%		3	7%	*p* = 0.012
Age (mean ±*sd*)		36.2 ± 11.9			38.9 ± 13.1		*p* = 0.342
Non-german		114	24%		5	13%	*p* = 0.118
Pre-trial		256	53%		7	18%	*p* < 0.001
Violent crime		248	52%		28	70%	*p* = 0.032
Mental disorder		32	7%		40	100%	*p* < 0.0001
Former detention	*N* = 314	120	38%	*N* = 35	9	26%	*p* = 0.195
Former suicide attempt		70	15%		19	48%	*p* < 0.0001

## Results

A total of 53 from 63 (84%) forensic institutions completed the questionnaire, while all German prisons (100%) participated in the survey. In total, the sample consisted of 519 completed suicide events: 479 prison suicides and 40 suicides in forensic psychiatric hospitals (see Table 1). The mean age of the group was 36.4 ± 12.0. Men committed 99% of the suicides, 23% were of non-German nationality, 51% were in an early stage of confinement, and 53% were in prison or in a forensic hospital because of a violent crime. In only 14% of the cases was a mental disorder detectable, and in 17% of the cases, former suicide attempts were documented. Thirty-seven percent of the people who died by suicide were in prison or in a forensic hospital before: thus, 63% were in prison or a forensic hospital for the first time. There was no statistical difference regarding mean age and the nationality in the two institutional settings. In prison, 53% of the suicide events occurred during pre-trial status, yet this was the case in (only) 18% of the suicide events in the forensic psychiatric hospitals (*p* < 0.0001). In 52% of the suicide events in prison, the reason for detention was a violent offense, while this was the case in 70% of the suicide events in the forensic psychiatric hospitals.

While all patients were diagnosed with at least one mental disorder (e.g., schizophrenia, personality disorder, substance abuse disorder) in the forensic psychiatric hospital, this was reported in only 7% in the group of prison suicides. In addition, former suicide attempts in the prison group were documented for 15% of the inmates who committed suicide, while this was known for 48% of the patients who committed suicide in the forensic setting. There was no statistical difference regarding the item of “former detention”.

The multivariate logistic regression analysis found significant effects for gender (*p* = 0.005, 95% confidence interval: [1.984, 140.583]), pre-trial status (*p* < 0.001, 95% confidence interval: [2.168, 17.374]), and former suicide attempt (*p* < 0.001, 95% confidence interval: [2.440, 11.892]).

**Figure 1:**
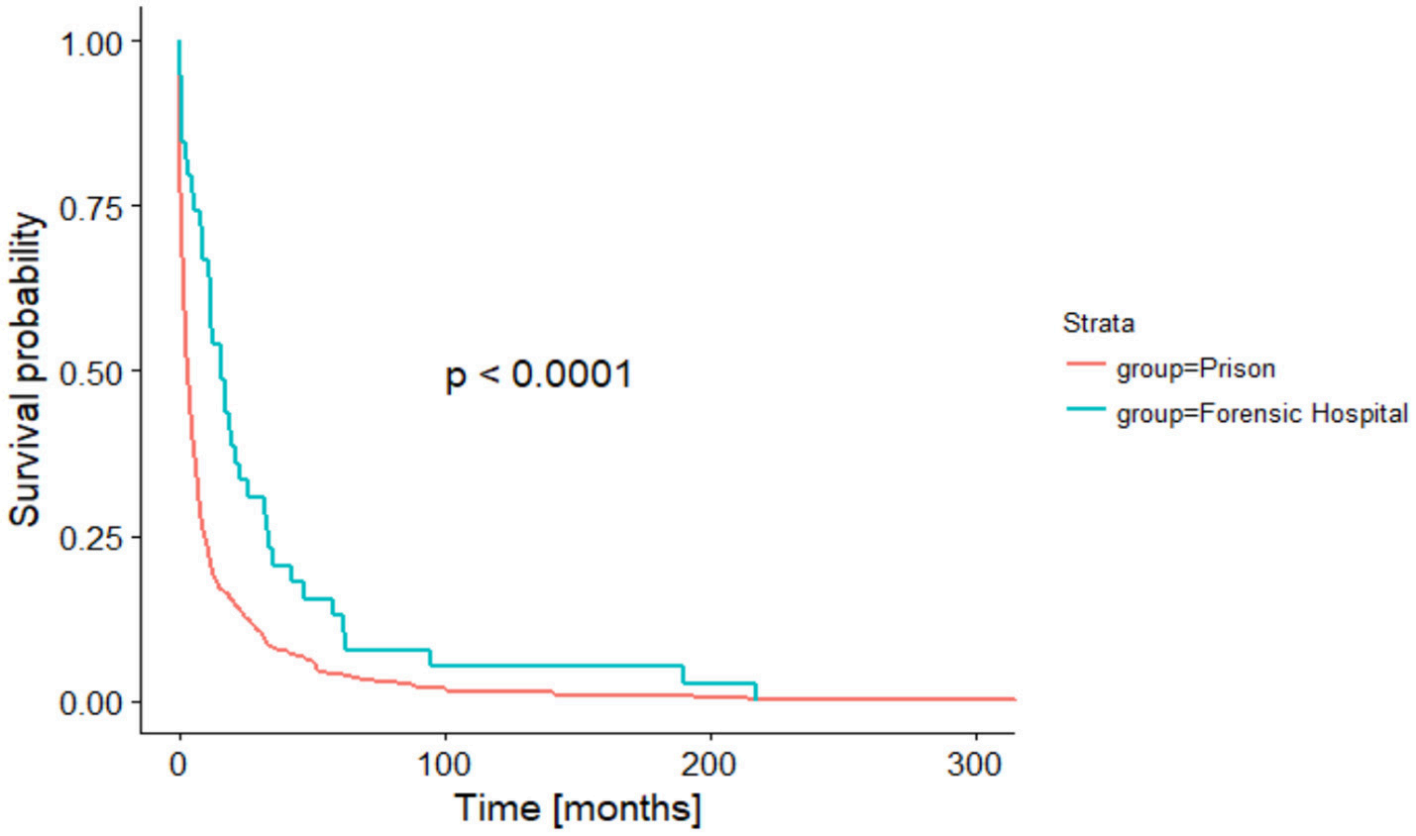
Time in month until suicide (Kaplan-Meier analysis).

The duration from admission into the institution to the suicide event was statistically significantly (*p* < 0.0001) shorter in the prison group in comparison to the forensic hospital group (see Figure 1).

**Figure 2:**
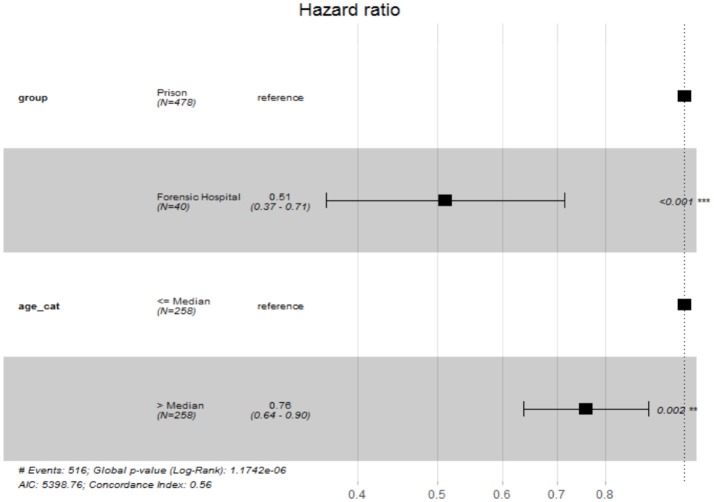
Hazard ratios of independent variables of Cox-Regression.

**Figure 3:**
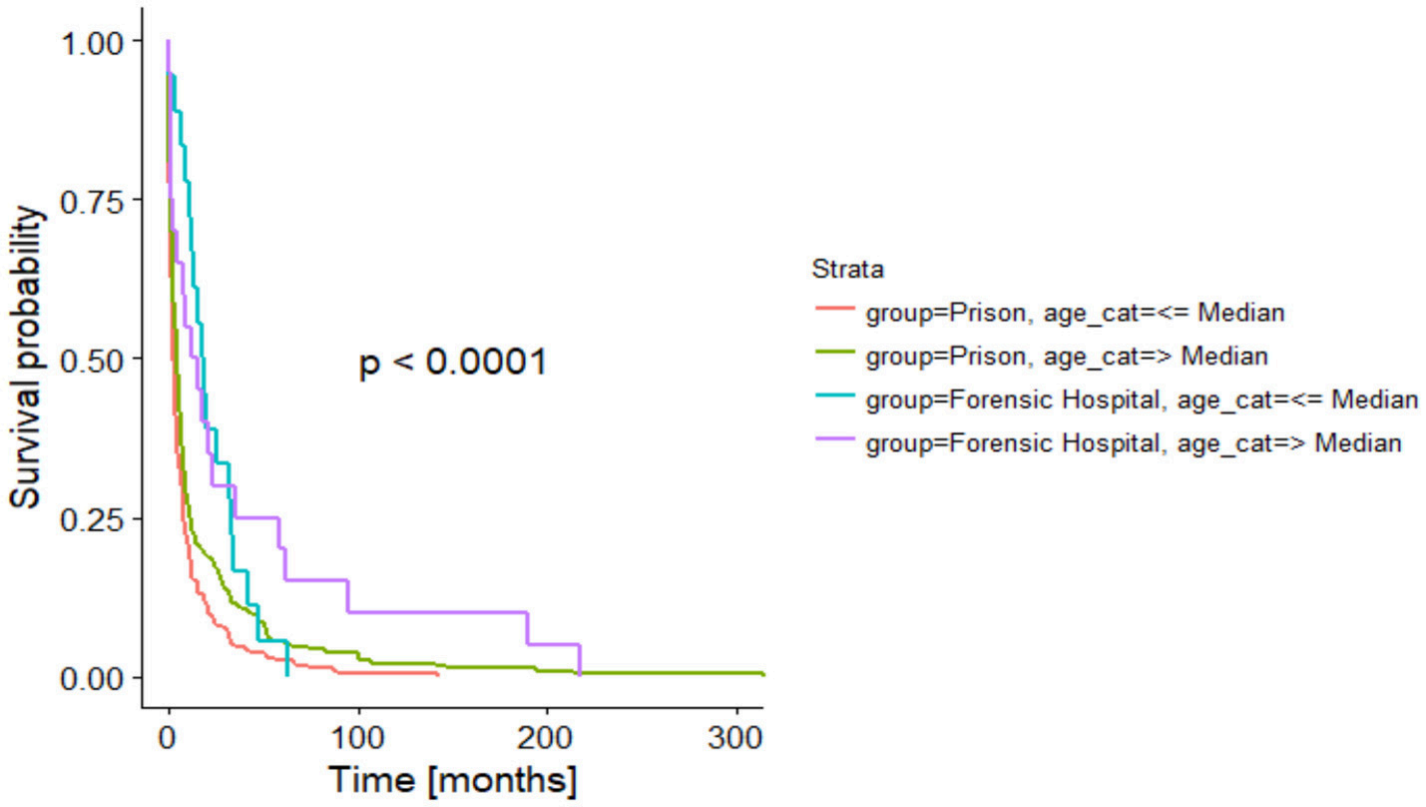
Time in months until suicide regarding age (Kaplan-Meier analysis).

After adjusting the time-to-event analysis for the age categories, we still found a significant difference between the two groups (*p* < 0.001) and a significant effect for age (*p* < 0.001), indicating that younger people tend to commit suicide earlier during their stay in a forensic psychiatric hospital or prison (see Figures 2, 3).

The mean suicide rate in the forensic psychiatric hospitals was 123/100,000 patients per year (95% confidence interval: [0.00103, 0.00147]). In comparison, the rate in the prison system was 130/100,000 prisoners per year (95% confidence interval: [0.00109, 0.00154]). This difference is not statistically significant (*p* = 0.706) (see Table 2).

**Table 2:** Suicide rate in Prisons and Forensic psychiatric hospitals in Germany.

	**Prisons**		**Forensic hospitals**	
	**Total pop**.	**Suicides**	**Total pop**.	**Suicides**
2000	70.252	117	5.617	4
2001	70.203	107	5.903	11
2002	70.977	77	6.587	7
2003	79.153	83	6.959	10
2004	79.452	95	7.278	8
Mean rate	130/100.000		123/100.000	

## Discussion

When we compared suicide events in the German institutional penal systems, we found that the mean suicide rates in the prison system and in the forensic hospitals did not differ statistically significant. Our hypothesis that a possible accumulation of general and specific risk factors in forensic psychiatric patients, as mentioned above, may lead to a higher rate of suicide events compared to the group of prisoners did not stand ground. However, an accumulation of general and specific risk factors were identified in the suicide events in the forensic hospitals: in all cases, a mental disorder was diagnosed, in 70% of the events, the patient was institutionalized due to a violent (including sexual) offense and in 48% of the events, former suicide attempts were known.

It is not surprising that most of the cases of reported suicide events in our study were committed by men. First, the male gender is an established risk factor (33), and second, in both institutional systems, the total populations consisted of a significantly higher proportion of men. There was no age difference in the groups nationwide. How the mean age differed from the total populations in both systems was not recorded.

In both groups, then majority of suicide events were commited by German citizens. Compared to the prison group, the proportion of non-German patients who committed suicide in forensic psychiatric hospitals was smaller, but this difference was not statistically significant. We didn't find comparable data regarding suicidality in forensic psychiatric settings, specifically regarding nationality.

It is interesting that the proportion of suicide events during pre-trial detention was significantly higher in the prison group (53 vs. 18%), which matches the findings of Bennefeld-Kersten (26) that pre-trial detention is a specific risk factor for prison suicide. In contrast, in the forensic psychiatric hospital, the pre-trial status of the patient seemed to be of lesser significance for suicidality. It seems understandable that in a specialized psychiatric setting, such as in the German forensic hospitals, suicide prevention is a routine task and due to professionally trained personnel (medical doctors/psychotherapists), treatment is optimized in comparison to a prison system.

The duration of stay from the admission to the suicide event differed significantly in both groups. Suicide events occurred earlier in prison than in forensic hospitals. In addition, in both institutions, the younger inmates and patients committed suicide earlier than older ones. Thus, young age could be understood as a potential risk factor for early suicide in forensic hospitals and in prison. It may be discussed if the later onset of suicide events in the forensic hospitals correlates with the distribution of diagnoses in the groups. Suicide risk in men with schizophrenia increases with the number of admissions to a hospital and thus over the course of time (34), while in patients with depression the suicidal risk decreases over the course of time, and suicide attempts are committed instead at the beginning of a depressive episode (35, 36). Another reason for the difference in time from the admission until the committed suicide could be the potentially unlimited duration of stay in German forensic psychiatric hospitals. The missing prospect of a final date of dismissal may lead to a feeling of hopelessness that could increase in the course of past (unsuccessful) years of therapy. Lack of hope has been described as a general risk factor for suicidality (12). To what extent the amendment of the German penal code for institutionalization in a forensic hospital will improve the prospects of patients within a forensic institution remains to be seen. These amendments from April 2016 included a higher frequency of external expert witness reports and a stronger focus on proportionality between the committed offense, the duration of stay and the probability of severe violent offenses in the future.

There was a small amount of information available on potential mental disorders in the group of prison suicide events; in only 7% of the cases was a psychiatric disorder registered. This is in stark contrast to the international literature (3, 4, 22) that indicated a higher prevalence of mental disorders in prison in general and in suicide events specifically, such as depressive episodes and adjustment disorders (6). In addition, the information on “former suicide attempts” was documented in only 15% of the prison group in comparison to 48% of the forensic hospital group. It seems that these two items in the prison group were underreported and thus must be considered a major limitation of this study. However, this may indicate an expression of a lack of standardized psychiatric assessments and suggest an optimization of the information collection process regarding suicide prevention. Data on former suicide attempts in prisoners is especially significant since this risk factor is (even) more important than socio-demographic data and mental disorders (37).

In summary, institutional suicide events can be considered multifactorial in nature. While in a prison setting, suicide events tend to occur at an earlier stage of the secure confinement, it is important to consider the influence of specific life events as “imprisonment” and “conviction” on suicidal thoughts and impulses. The use of a specific screening tool for suicidality at the beginning of imprisonment, as well as during critical landmarks (verdict, trial days) consequently seems recommendable. Mental health in general, as well as the specific former psychiatric history, should be frequently addressed and documented by the personnel in charge. In forensic psychiatric hospitals, because of the plentitude of patients with schizophrenia, the natural (and often chronic and complicated) course of the disorder must be considered when interpreting the results. Apart from the higher suicide risk within this disorder itself, a possible lack of future prospects due to the uncertain duration of stay may relate to a higher suicide rate and later onset of suicide events in forensic psychiatric institutions. This should be a possible starting point for further research activities aiming for an optimization of suicide prevention in forensic institutions.

Our study has a number of significant limitations. It was not possible to compare our findings with the total numbers within each institution separately, as this data was not obtainable. In addition, the presented numbers in the groups were very small and, in respect to certain items, incomplete (“former detention”). The data concerning mental disorders in prison seemed to be vastly underreported, which makes a comparison with inmates of a forensic psychiatric hospital, where every patient has a mental disorder, especially difficult.

## Ethics statement

According to current legal regulation, no approval from the local ethics committee was required for the current study.

## Author contributions

AV and AO-W designed the study. AV, NaK, and AO-W collected the data. AV, NK, and AO-W analyzed and interpreted the data. AV and AO-W wrote the initial draft of the manuscript. AV and AO-W had full access to all the data in the study and take responsibility for the integrity of the data and the accuracy of data analysis. All authors have contributed to, read, and approved the final version of the manuscript.

### Conflict of interest statement

The authors declare that the research was conducted in the absence of any commercial or financial relationships that could be construed as a potential conflict of interest.
